# Increased TLR4 Expression Aggravates Sepsis by Promoting IFN-*γ* Expression in CD38^−/−^ Mice

**DOI:** 10.1155/2019/3737890

**Published:** 2019-02-19

**Authors:** Qi Li, Chenyi Wu, Zhenlong Liu, Huiqing Zhang, Yuna Du, Yuxiang Liu, Kuangyu Song, Qiaofa Shi, Rong Li

**Affiliations:** ^1^Department of Medical Microbiology & Immunology and Laboratory of Infection & Immunity, School of Basic Medical Sciences, Nanchang University, Nanchang 330006, China; ^2^McGill University, Division of Experimental Medicine, Department of Medicine, Montreal, Quebec, Canada

## Abstract

Gram-negative bacterial sepsis accounts for up to 50% worldwide sepsis that causes hospital mortality. Acute kidney injury (AKI), a common complication of Gram-negative bacterial sepsis, is caused by Toll-like receptor 4 (TLR4) activation. Lipopolysaccharide (LPS) is an endotoxin in Gram-negative bacteria and is recognized specifically by TLR4, which initiates innate immune response. Also, TLR4 signaling pathway activation is essential in response to LPS infection. CD38 is one of the well-known regulators of innate immunity, whose dysregulation contributes to sepsis. Many studies have proven that an attenuated Gram-positive bacterium induces sepsis in a CD38-blocking model. However, the pathogenesis of Gram-negative bacteria-induced sepsis in a CD38^−/−^ mouse model remains unclear. The aim of this study is to investigate whether kidney injury is still attenuated in a LPS-induced CD38^−/−^ sepsis model and identify the potential mechanism. We assess the severity of kidney injury related to proinflammatory cytokine expressions (IFN-*γ*, TNF-*α*, IL-1*β*, and IL-6) in WT and CD38^−/−^ mice. Our results showed more aggravated kidney damage in CD38^−/−^ mice than in WT mice, accompanied with an increase of proinflammatory cytokine expression. In addition, compared with CD38^−/−^TLR4^mut^ mice, we found an increase of TLR4 expression and mRNA expression of these cytokines in the kidney of CD38^−/−^ mice, although only increased IFN-*γ* level was detected in the serum. Taken together, these results demonstrated that an increased TLR4 expression in CD38^−/−^ mice could contribute to the aggravation of AKI through boosting of the production of IFN-*γ*.

## 1. Introduction

Sepsis, a well-known systemic inflammatory response to various infections [1, 2], is life-threatening due to multiorgan dysfunction resulting from the dysregulation of host response to infection [3]. Innate immunity conducts itself as the first line of host defense against infection, which plays a key role in the development of sepsis [4]. Many studies have already proved that an abnormal innate immune response contributes to sepsis-induced organ dysfunction, where excessive inflammatory responses such as cytokinaemia and excessive neutrophils infiltration lead to host cell and tissue damage, and ultimately proceed to organ failure [5–7]. Furthermore, IFN-*γ* has been indicated as a predominant cytokine produced during innate immune responses, promoting aggravated sepsis [8–10].

IFN-*γ* is a type II interferon, which can initiate immune responses against infection [9]. Despite its well-known role in inhibiting viral replication, as a proinflammatory cytokine, IFN-*γ* also functions to activate immune cells like natural killer cells and macrophages [8]. In addition, IFN-*γ* enhances the ability of infected host cells to process antigen by promoting antigen presentation [11, 12]. Studies have proved that upregulation of IFN-*γ* results in a more severe sepsis syndrome [8, 13]. Moreover, in cecal ligation and a puncture-induced sepsis mouse model, mice suffered from aggravated sepsis due to an excess of IFN-*γ* released by activated natural killer T cells (NKT cells), while mice with deficiency in IFN-*γ*-producing NKT cells have a higher survival rate when encountering sepsis challenge [8, 14, 15]. Additionally, NKT cells are also proven to take part in autoimmune diseases [16–18] and known to produce large amounts of IFN-*γ* rapidly when activated to regulate innate immune response against infection [16]. NKT cells promote arthritis in mice induced by antibodies through cytokine production which involved IFN-*γ* [18]. IFN-*γ*-producing NKT cells were shown to promote septic shock syndrome [19]. These all suggests that IFN-*γ* is one of the important components that contribute to severe sepsis condition.

Most sepsis cases are caused by bacterial infection [1, 6]. Although Gram-positive bacterial sepsis remains the most prevalent worldwide, Gram-negative bacterial sepsis accounted for 50% of sepsis cases in China [20]. We focused on investigating sepsis induced by lipopolysaccharide (LPS), which is an endotoxin of the Gram-negative bacteria. LPS is partly able to trigger a rapid innate immune response through facilitating the activation of IFN-*γ* in NKT cells that directly clear up the infection [9]. However, when the following inflammatory response is in excess, host cells and tissues will get damaged. Ogasawara et al. showed that in the immediate phase of immune response to LPS stimuli, the subsequent upregulation of IFN-*γ* could promote liver injury, septic shock, and even death [21].

TLR4 is confirmed to recognize LPS [5, 22], and it regulates the production of proinflammatory cytokines such as tumor necrosis factor-*α* (TNF-*α*) and interleukin-1 beta (IL-1*β*) [5] and mediates infiltration and activation of inflammatory cells to respond to infectious pathogens [22]. As TLR4 expresses on the renal tubular epithelium, glomeruli, and vascular endothelium, it was assumed to play an important role in sepsis-induced AKI [22], which was characterized by tubular injury including tubular epithelial cell edema, interstitial edema, and tubular lumen narrowing, or with glomerular injury such as swelling of glomerular epithelial cells, narrowing of the glomerular capillary lumen, and glomerular congestion [22]. By employing TLR4^−/−^ mice [23–25] as the model, more evidence has shown that blocking TLR4 protects mice from severe kidney damage in LPS-induced sepsis. TLR4 activation in response to LPS stimuli will cause AKI where the normal functions of both renal tubule and glomerulus are disrupted [22–25]. Kim et al. reported that TLR4 could be expressed both on the surface and in the endosome of NKT cells [16]. Also, TLR4 was identified as an indirect activator of NKT cells through TLR4-dependent activation of APCs [26]. Furthermore, an intriguing study provided evidence that TLR4 can directly activate NKT cells to regulate immune response through IFN-*γ* production [16].

CD38 is a transmembrane glycoprotein expressed on the surface of both myeloid and lymphoid cells, including NK cells and some T cell subtypes [27, 28]. CD38 functions as both cyclase and hydrolase which regulates intracellular calcium release [29], extracellular Ca^2+^ influx [27], and chemotaxis [30]. Studies indicated that CD38^−/−^ mice have an impaired innate immunity including ineffective recruitment of neutrophils and monocytes towards sites of infection [27, 28, 31]. Since dysregulated innate immune response largely contributes to sepsis [9], we turned our attention to CD38, an important regulator of innate immunity [27]. Moreover, Musso et al. suggested that LPS stimuli could not upregulate CD38 expression alone, while IFN-*γ* instead can strongly stimulate the expression of CD38 on monocytes and myeloid cell lines via a dose-dependent manner. However, in a CD38-deficient myeloid cell line, IFN-*γ* is unable to induce CD38 expression [12].

Therefore, we investigated in CD38^−/−^ mice whether IFN-*γ* is still a dangerous factor that promotes sepsis progression induced by LPS challenge, with the aim of indicating a potential therapeutic field to overcome sepsis. We had two hypotheses; one was that CD38 knockout would impair the stronger innate immune response in sepsis, thus resulting in an attenuated sepsis syndrome with a less severe kidney damage. Based on previous studies, TLR4 activation has great significance in Gram-negative sepsis-induced kidney injury [22, 32], and TLR4 can activate the NKT cell which is a dominant IFN-*γ* production immune cell [16]. The other hypothesis was that a more serious kidney injury would be present in CD38^−/−^ mice, ascribing the TLR4 role in promoting IFN-*γ* expression. To test our hypotheses, we observed the severity of kidney injury and examined inflammatory cytokine expression in WT and CD38^−/−^ mice 2 hours post-LPS infection. Furthermore, we constructed a CD38^−/−^TLR4^mut^ mouse model to study the effects of TLR4 in regulating immune responses in CD38^−/−^ mice in response to LPS stimuli.

## 2. Materials and Methods

### 2.1. Animals

Specific pathogen-free wild-type (C57BL/6) mice were purchased from the Laboratory Animal Center of Wuhan University at 4 weeks of age. CD38^−/−^ mice (B6.129P2-Ly96^−/−^) and CD38^−/−^TLR4^mut^ mice (other than TLR4^−/−^ mice in which TLR4 is totally knocked out, while TLR4^mut^ mice is the mice with the mutation of the LPS target site on TLR4) were kindly offered by Professor Hongbo Xin and Keyu Deng in the Translational Medicine Research Institute of Nanchang University. These three different types of mice were grouped into cages according to the same female-male ratio of 2 : 1, and the mice were fed in the SPF Animal Facility at the Laboratory Animal Center of Nanchang University. Male mice were selected for use at the age of 8 weeks. All experiments conformed to the Management Ordinance of Laboratory Animals of Nanchang University and were approved by the Animal Protection Committee of Nanchang University.

To create a Gram-negative bacterial sepsis mouse model, mice were injected intraperitoneally (i.p.) with LPS 20 mg/kg body weight as reported previously [4, 33]. And according to Wang et al. [34], “proinflammatory gene expressions were significantly increased at both 2 hours and 8 hours after LPS injection, and expression of cytokine levels after 2 hours were significantly higher than that after 8h of LPS injection”; we sacrificed the mice 2 hours after LPS injection for the following study.

### 2.2. Body Weight Measuring and Analyzing

The weight of the mice was weighed and recorded before and 2 hours after LPS injection. The percentage of weight loss was calculated as an indicator of weight change. And to make sure that the change of body weight was only induced by LPS stimuli, the mice were supplied without any food or water during the 2 hours.

### 2.3. Histological Staining

The mice were sacrificed 2 hours after LPS injection. The kidneys from both the WT and CD38^−/−^ mice were removed and the kidney tissues were fixed in 4% (wt/vol) paraformaldehyde (PFA) overnight, and then washed with 0.1 M PBS (pH 7.4) 3 times. The kidney tissues were dehydrated in a gradient of alcohols (50%, 75%, 95%, and 100% twice) subsequently, and then processed for paraffin embedding by classic procedure. Serial paraffin sections throughout the kidney were cut into 3 *μ*m thickness on a microtome and heated at 65°C for 2 hours for deparaffinization. Hydration was done by transferring the sections through the following solutions: triple to xylene for 5 mins, and then for 30 secs to 100% ethanol twice, 95% ethanol, and 70% ethanol. Sections were stained with H&E according to the manufacturer protocol and mounted on glass slides.

### 2.4. RNA Extraction and Real-Time Quantitative PCR Assay

Total RNA was isolated from the kidney tissues (50-100 mg) using TRIzol (Invitrogen, US). Then 1 *μ*g RNA was extracted and further treated with gDNA Eraser (Takara Biotechnology Company) at 42°C for 2 minutes, for the purpose of removing gDNA. The prepared RNA was therefore ready for RT-PCR with the help of the PrimeScript™ RT. RT-PCR conducted at an initial 15 min at 37°C, then 5 sec at 85°C, ended up with 4°C for product preservation.

RT-qPCR was then performed using the SYBR® Premix Ex Taq™ II (Tli RNaseH Plus) and carried out using the StepOnePlus™ Real-Time PCR System (Applied Biosystems, New York, USA). Primers of genes including TNF-*α*, IL-6, IL-1*β*, IFN-*γ*, TLR4, and GAPDH were synthesized from Sangon Biotech (Sangon Biotech, Shanghai, China) (the GADPH forward primer, 5′-GAA GGT GGT GAA GCA GGC ATC; the GADPH reverse primer, 5′-GTG GGA GTT GCT GTT GAA GTC; the IL-1*β* forward primer, 5′-TTT TCC TCC TTG CCT CTG AT-3′; the IL-1*β* reverse primer, 5′-GAG TGC TGC CTA ATG TCC CC-3′; the TNF-*α* forward primer, 5′-AGC GGA TGG GTT GTA CCT TG-3′; the TNF-*α* reverse primer, 5′-GTG GGT GAG GAG CAC GTA GTC-3′; the IFN-*γ* forward primer, 5′- CAG GCC ATC AGC AAC AAC ATA AGC-3′; the IFN-*γ* reverse primer, 5′- AGC TGG TGG ACC ACT CGG ATG-3′; the IL-6 forward primer, 5′- ACT TCC ATC CAG TTG CCT TCT TGG-3′; the IL-6 reverse primer, 5′- TTA AGC CTC CGA CTT GTG AAG TGG-3′; the TLR4 forward primer, 5′- CGC TTT CAC CTC TGC CTT CAC-3′; and the TLR4 reverse primer, 5′- TTG CCG TTT CTT GTT CTT CTT C-3′). PCR conditions consisted of 30 s at 95°C, followed by 40 cycles of 5 s at 95°C, and 30 s at 60°C. Moreover, the threshold cycle (Ct) was detected for each gene, and the levels of gene expression relative to GADPH were determined.

### 2.5. Enzyme-Linked Immunosorbent Assay (ELISA)

To measure the cytokine levels in a mouse serum, cytokine-specific ELISA kits (eBioscience) were used according to the manufacturer's instructions. The total amount of TNF-*α*, IL-6, IL-1*β*, and IFN-*γ* in the serum was normalized to the total amount of protein in the viable cell pellets. The signal was detected by using a microplate reader set at 450 nm with the correction of 570 nm.

### 2.6. Western Blot Analysis

To further determine the change of TLR4 expression in the mouse kidney, the kidney tissues from the LPS-stimulated WT, CD38^−/−^, and CD38^−/−^TLR4^mut^ mice (*n* = 3/group) were squashed and lysed in the RIPA Lysis Buffer. An equal amount of protein was loaded into SDS-PAGE and transferred onto polyvinylidene difluoride membranes. The primary antibodies used are anti-TLR4 Rabbit mAb (1 : 500) (Proteintech, USA) and anti-GAPDH Mouse mAb (1 : 5000) (CST, USA). The expression of TLR4 and GAPDH was visualized by the ECL assay (Sagecreation) according to the manufacturer's instructions.

### 2.7. Immunohistochemical Staining

For immunostainings, sections were deparaffinized, rehydrated in graded alcohols successively, and then blocked by incubating in 0.3% H_2_O_2_ for 10 min. The slides were incubated with primary antibodies of anti-TLR4 Mouse mAb (1 : 400) (Santa Cruz, USA), anti-NF-*κ*B p65 Rabbit mAb (1 : 800) (CST, USA), and anti-NF-*κ*B1 p105 Rabbit mAb (1 : 500) (Abcam, USA) for 50 min at room temperature. After a thorough wash in phosphate-buffered saline (PBS), the tissue slides were incubated with biotin-conjugated secondary antibodies for 25 min at room temperature. The signal was detected using diaminobenzidine (DAB).

### 2.8. Statistical Analysis

All data were expressed as means ± standard deviation (SD). The paired *t*-test was used to determine statistically significant differences between the different groups. Differences were considered significant when *p* values were <0.05. The statistical software used was GraphPad Pro 5.0 (GraphPad, San Diego, CA, USA).

## 3. Results

### 3.1. CD38^−/−^ Mice Show Signs of Severe Illness according to LPS Infection, and the Degree of Weight Loss Is Significant Compared with the WT Mice

**Figure 1:**
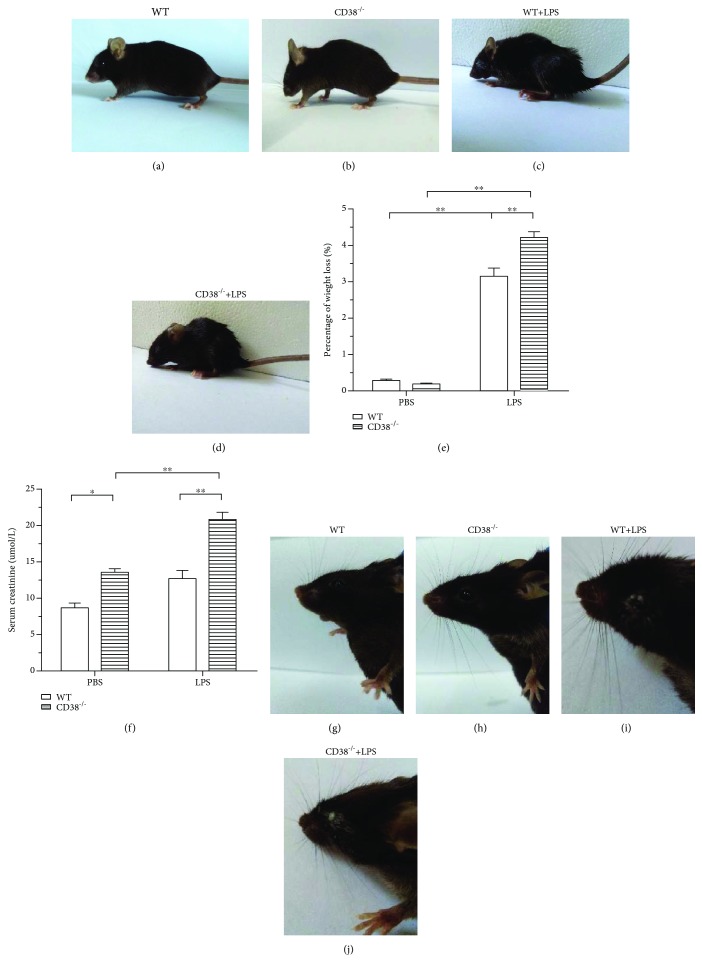
The LPS infection which induced changes in appearance and weight were more serious in CD38^−/−^ mice. (a–d) 2 hours post-LPS injection, mouse showed signs of illness in appearance. (e) The percentage of sepsis mouse weight loss 2 hours after LPS infection, WT and CD38^−/−^ mice, each group contains 5 male mice. (f) The serum creatinine (Scr) level in the WT and CD38^−/−^ mice 2 hours after PBS or LPS injection. (g–j) CD38^−/−^ mouse represented more evident encrusted eye. Data are presented as means ± standard deviation (SD). Statistical significance was determined by the paired *t*-test. *n* = 10, ^∗∗^*p* < 0.01.

To investigate the potential roles of CD38 in LPS-challenged sepsis, we observed the phenotype change in a CD38^−/−^ mouse model. We intraperitoneally infected the WT and CD38^−/−^ mice with 20 mg/kg body weight LPS. Two hours postinfection, we found that both the WT and CD38^−/−^ mice show signs of weakness such as rough fur and encrusted eyes (Figures 1(a)–1(d)). And compared to the WT mice, the CD38^−/−^ mice were more susceptible to LPS and showed a much bleaker color in fur. 2 hours after LPS injection, although the WT mice lost 3% of their body weight, the CD38^−/−^ mice lost more than 4% of their body weight. Meanwhile, the WT mice and the CD38^−/−^ mice without LPS injection only underwent 0.3% and 0.2% weight loss, respectively (Figure 1(e)). The serum creatinine was increased after LPS stimuli, and this increase was higher in the CD38^−/−^ mice 2 hours after LPS injection (Figure 1(f)). In addition, compared to the WT mice, the CD38^−/−^ mice seemed to undergo more severe illness in appearance, which was particularly reflected by the more obvious encrusted eyes (Figures 1(g)–1(j)). All the differences above showed that the CD38^−/−^ mice have suffered a more serious LPS-induced infection than the WT mice.

### 3.2. Kidney Injury Is More Severe in the CD38^−/−^ Mice Caused by the Activation of TLR4

**Figure 2:**
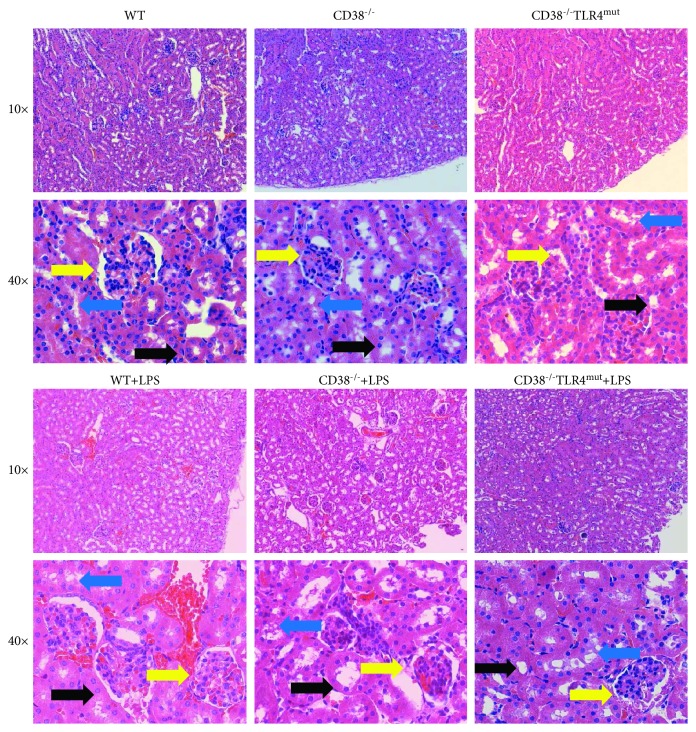
CD38^−/−^ mice show a stronger inflammatory response and suffer from more severe kidney damage than WT mice according to LPS injection by the TLR4 pathway. The mice were injected with LPS (20 mg/kg) and sacrificed after 2 hours after injection. Representative hematoxylin and eosin staining images. The yellow arrow indicates narrowing of the glomerular capillary lumen and glomerular congestion, the black arrow indicates the tubular epithelial cell edema, and the blue arrow indicates interstitial edema.

We compared the severity of kidney injury between the CD38^−/−^ and WT mice. We found that there is a stronger inflammatory response and a corresponding severe kidney injury in the CD38^−/−^ mice. We observed a serious infiltration of inflammatory cells, tubular epithelial cell edema, interstitial edema, and tubular lumen narrowing in the CD38^−/−^ mice, whereas these changes in the kidneys of the CD38^−/−^ mice were rescued in the CD38^−/−^TLR4^mut^ mice. And these showed a stronger inflammatory reaction in response to LPS challenge in the CD38^−/−^ mice by the activation of the TLR4 pathway (Figure 2).

### 3.3. A Distinct Increased Proinflammatory Cytokine mRNA Expression Was Observed in the Kidneys of the CD38^−/−^ Mice Compared with the WT Mice

**Figure 3:**
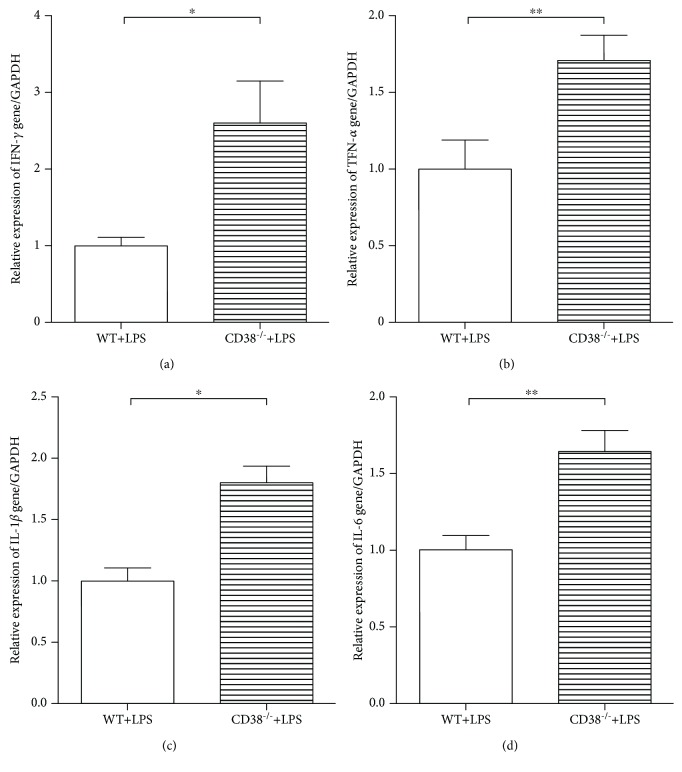
CD38^−/−^ mice have a stronger inflammatory response to LPS challenge. The mRNA of the kidney proinflammatory cytokines (a) IFN-*γ*, (b) TNF-*α*, (c) IL-1*β*, and (d) IL-6 of the WT and CD38^−/−^ mice were measured by RT-qPCR 2 hours after LPS stimuli. *n* = 10, ^∗^*p* < 0.05, ^∗∗^*p* < 0.01.

To try to explain the aggravated kidney injury in the CD38^−/−^ mice, we then tested the levels of all the following kidney proinflammatory cytokine mRNA: IFN-*γ* (Figure 3(a)), TNF-*α* (Figure 3(b)), IL-1*β* (Figure 3(c)), and IL-6 (Figure 3(d)) mRNA. These data demonstrated a notably higher expression of all these cytokines in the CD38^−/−^ mice than the WT mice 2 hours after LPS challenge, suggesting a stronger inflammatory response in the CD38^−/−^ mice.

### 3.4. A Momentous Growth of Expression Levels of Serum Proinflammatory Cytokines Were Observed in the CD38^−/−^ Mice Compared with the WT Mice

**Figure 4:**
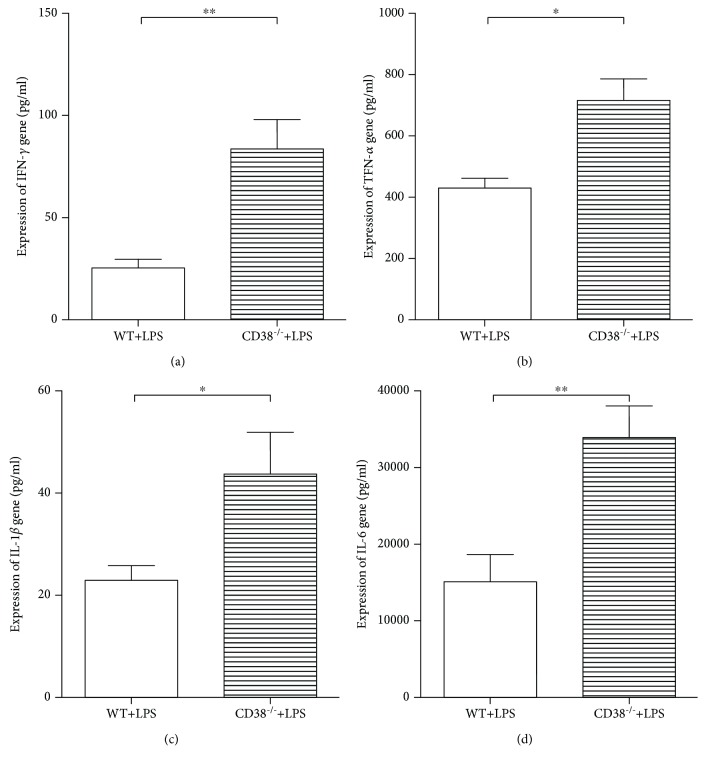
Serum proinflammatory cytokine expression was upregulated in the CD38^−/−^ mice. (a) IFN-*γ*, (b) TNF-*α*, (c) IL-1*β*, and (d) IL-6 expressions in serum were measured by ELISA 2 hours post-LPS injection. Data are presented as means ± standard deviation (SD). Statistical significance was determined by the paired *t*-test. *n* = 10, ^∗^*p* < 0.05, ^∗∗^*p* < 0.01.

To further confirm the increase of proinflammatory cytokine expression, we then measured the expression levels of the above cytokines in serum by ELISA. Interestingly, compared to the WT mice, the consequential elevated expression of IFN-*γ* (Figure 4(a)), TNF-*α* (Figure 4(b)), IL-1*β* (Figure 4(c)), and IL-6 (Figure 4(d)) was observed in the CD38^−/−^ mice.

### 3.5. Increased TLR4 Expression in the CD38^−/−^ Mice Causes an Aggravated Kidney Injury in LPS-Induced Sepsis through Promotion of IFN-*γ* Expression

**Figure 5:**
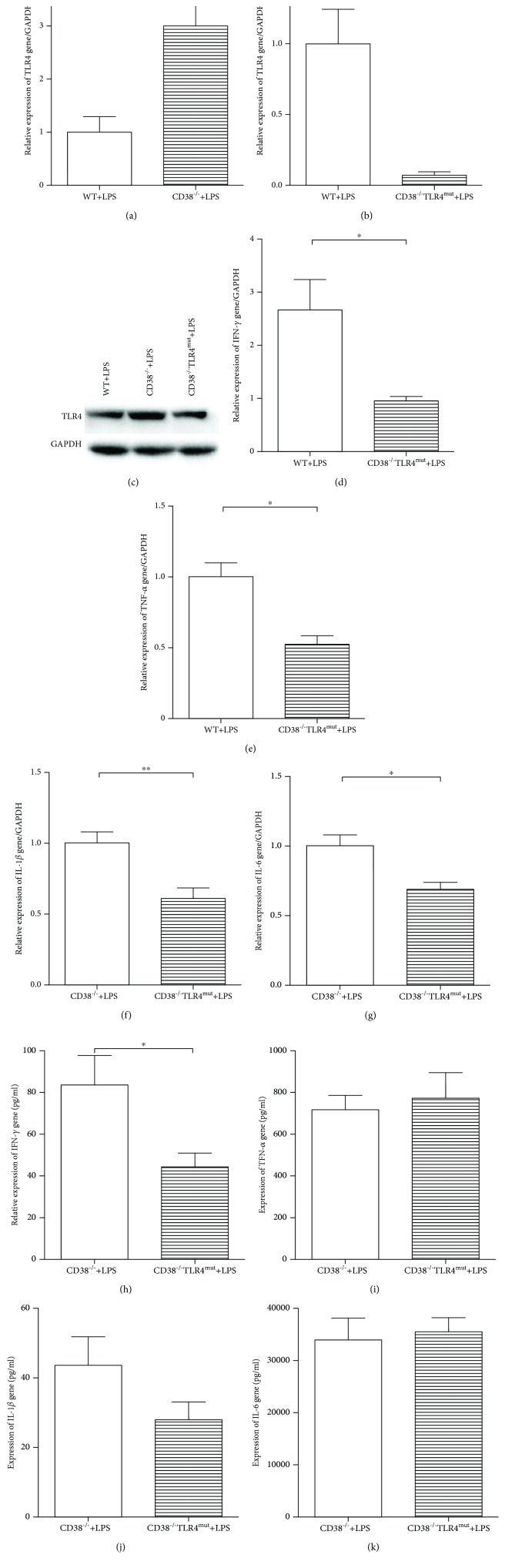
The elevated expression of mRNA of proinflammatory cytokines and increased expression of IFN-*γ* in serum induced by LPS challenge in CD38^−/−^ mice were rescued in the CD38^−/−^TLR4^mut^ mice. Expression of TLR4 in the kidney was measured 2 hours after LPS injection. (a) A significant increased expression of mRNA level of TLR4 in the CD38^−/−^ mice. (b) A significant decreased expression of mRNA level of TLR4 in the CD38^−/−^TLR4^mut^ mice. (c) Expression of protein level of TLR4 is increased significantly in the kidney of the CD38^−/−^ mice and decreased in the kidney of the CD38^−/−^TLR4^mut^ mice. Changes in proinflammatory cytokine expression in the CD38^−/−^TLR4^mut^ mice compared with the CD38^−/−^ mice two hours after LPS injection. (d–g) Expression of IFN-*γ*, TNF-*α*, IL-1*β*, and IL-6 mRNA in the kidney. (h–k) Expression of IFN-*γ*, TNF-*α*, IL-1*β*, and IL-6 in serum. Data are presented as means ± standard deviation (SD). Statistical significance was determined by the unpaired *t*-test. *n* = 10, ^∗^*p* < 0.05, ^∗∗^*p* < 0.01.

After TLR4 recognizes LPS infection, it initiates immune reactions to try to clear up the infection, for instance by promoting the production of proinflammatory cytokines [5] and mediating infiltration and activation of inflammatory cells [22]. We thus investigated the regulating capability of TLR4 in aggravating LPS-induced kidney injury in the CD38^−/−^ mice. Firstly, TLR4 levels were measured and showed a large increase in the CD38^−/−^ mice 2 hours after LPS injection (Figures 5(a) and 5(c)). To further explore the mechanism of TLR4 in prompting more severe kidney injury in the CD38^−/−^ mice, we developed a CD38^−/−^TLR4^mut^ mouse model where the TLR4 expression should be kept in a relative low level in response to LPS stimuli. To verify the model, we firstly stimulated both the CD38^−/−^TLR4^mut^ and CD38^−/−^ mice with 20 mg/kg BW LPS and compared the TLR4 expression two hours later. We tested a dramatic decreasing of TLR4 expression level in the CD38^−/−^TLR4^mut^ mice as expected (Figures 5(b) and 5(c)). To study the effects of TLR4 on proinflammatory cytokine production, renal IFN-*γ*, TNF-*α*, IL-1*β*, and IL-6 mRNA expressions 2 hours after LPS injection were measured, and the results showed that all these cytokines consequentially decreased in the CD38^−/−^TLR4^mut^ mice (Figures 5(d)–5(g)). However, in the serum, only the IFN-*γ* expression was downregulated in the CD38^−/−^TLR4^mut^ mice compared with the CD38^−/−^ mice (Figures 5(h)–5(k)). Although the elevated expression of mRNA of proinflammatory cytokines induced by LPS challenge in the CD38^−/−^ mice was rescued in CD38^−/−^TLR4^mut^ mice, we only detected the rescued serum IFN-*γ* expression without change in the serum level of TNF-*α*, IL-1*β*, and IL-6. These reminded us that maybe IFN-*γ* is the key molecule in the LPS-induced sepsis pathway in the CD38^−/−^ mice. Taken together, upregulation of TLR4 in the CD38^−/−^ mice could cause an aggravated kidney injury in sepsis induced by LPS through promotion of the IFN-*γ* expression.

### 3.6. Increased TLR4 Expression in the CD38^−/−^ Mice Caused Migration of NF-*κ*B from the Cytoplasm to the Nucleus

**Figure 6:**
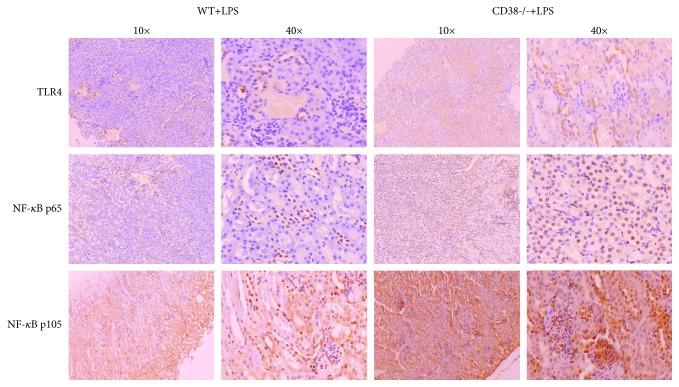
Effects of increased TLR4 expression on NF-*κ*B p65 and NF-*κ*B1 p105 localization: (top) expression of TLR4, (middle) nuclear translocation of NF-*κ*B p65, and (bottom) nuclear translocation of NF-*κ*B1 p105 (*n* = 3/group).

The TLR4-NF-*κ*B signal pathway plays an important role in the inflammatory process. So immunohistochemical staining for TLR4, NF-*κ*B p65, and NF-*κ*B1 p105 was used to show their expression and localization in the kidneys of the WT and CD38^−/−^ mice (Figure 6).

## 4. Discussion

In the present study, we demonstrated that CD38^−/−^ mice experienced a significant weight loss and a more severe kidney injury two hours after LPS (20 mg/kg BW) infection compared to WT mice. Our data showed that proinflammatory cytokines were upregulated in the CD38^−/−^ mice due to stronger inflammatory responses. In addition, we indicated that the upregulation of TLR4 in the CD38^−/−^ mice is able to promote the production of IFN-*γ,* thus enhancing inflammatory reactions which ultimately leads to aggravated sepsis.

The effects of blocking CD38 in bacterial sepsis is contradictory. Recent studies have reported that CD38 is essential in calcium mobilization [27]; in cell activation, proliferation, and differentiation [35]; and in regulating innate immune response [27]. Several evidences have already proved the role CD38 played in responding to Gram-positive bacterial infection. For example, an ineffective neutrophil [27] and macrophage [28] migration were observed in CD38^−/−^ mice when infected by *Streptococcus pneumoniae* and *Listeria monocytogenes*, respectively. This inflammatory cell infiltration deficiency results in an impaired innate immunity that renders CD38^−/−^ mice more vulnerable to Gram-positive bacterial infection. In addition, Peng et al. have revealed that by blocking the CD38/cADPR/Ca^2+^ pathway, heart, liver, lung, and kidney injuries were alleviated in the CLP surgery-induced sepsis mouse model [36]. Shu et al. showed that AKI was attenuated in an LPS-induced sepsis mouse model after CD38 was blocked by quercetin injected intraperitoneally [35]. Meanwhile, we found that CD38 gene deficiency could aggravate AKI in LPS-induced sepsis, which is not consistent with the reports above [27, 28, 35, 36]. The discrepancy might be due to the differences in the methods used to develop the sepsis model, for the TLR4 pathway was not activated in those sepses induced by Gram-positive bacteria [27] or polymicrobia [37]. In the LPS-induced Gram-negative bacterial sepsis model used in our study, the TLR4 pathway was activated to induce serious AKI.

TLR4 plays an essential role in promoting inflammatory response to LPS infection in CD38^−/−^ mice. TLR4 is expressed not only in certain immune cells but also in the renal tubule epithelium, glomeruli, and vascular epithelium [22]. It has been well studied that the activation pathway of TLR4 in response to LPS infection contributes to acute kidney injury [22]. LPS induces TLR4 dimerization through MD-2 which is an adapter protein [38, 39]. Some of the activated TLR4s are located on the plasma membrane, while the others are associated with CD14 (cluster of differentiation 14) forming TLR4/MD2/CD14 complexes internalized into endosomes [40]. The different locations of TLR4 determine the different downstream intracellular signaling pathways. The TLR4 complex located on a plasma membrane undergoes the TLR4/MyD88/NF-*κ*B pathway, while TLR4 in endosomes mediates the TLR4/TRIF/IRF3/IFN pathway [22, 40]. TLR4 is able to increase proinflammatory cytokine (TNF-*α*, IL-1*β*, and IL-6) expression through the NF-*κ*B pathway [41]. And the activated IRF3 can act as a transcription factor to induce IFN expression through TRIF-mediated phosphorylation of IRF3 [40]. Consistent with our CD38^−/−^ mouse model where TLR4 expression is elevated, we found higher mRNA expressions of TNF-*α*, IL-1*β*, IL-6, and IFN-*γ* in CD38^−/−^ mice than in WT mice after LPS challenge. And the increased expression of TLR4 is accompanied with the increased nuclear translocation of NF-*κ*B p65 and NF-*κ*B1 p105 in the kidneys of CD38^−/−^ mice. In addition, comparing CD38^−/−^TLR4^mut^ mice with CD38^−/−^ mice, we observed decreased mRNA expressions of TNF-*α*, IL-1*β*, IL-6, and IFN-*γ*. (TLR4^mut^ mice are the mice with the mutation of the LPS target site on TLR4, whereas TLR4^−/−^ mice mean the mice in which TLR4 is totally knocked out.) However, at the protein level, only the IFN-*γ* expression is significantly reduced. Thus, we conjectured that the increased TLR4 facilitates the expression of IFN-*γ* to promote inflammatory responses, thus causing more severe kidney injury induced by LPS in CD38^−/−^ mice.

Our finding indicates that specifically targeting and inhibiting the IFN-*γ* expression could be potential therapeutic strategies in the clinic to alleviate severe AKI in LPS-induced sepsis. It is intriguing to further investigate whether the increased kidney damage in CD38^−/−^ mice was caused by IFN-*γ* via using a blocking antibody to block IFN-*γ* production. Based on our data, moreover, CD38 deficiency can induce the expression of IFN-*γ*, which gives us a hint that CD38 may be a potential inhibitor of IFN-*γ*. Thus, we will also exploit CD38 agonists or CD38 signaling regulators, which have already shown an indispensable role in reducing response to LPS stimuli based on our study.

## 5. Conclusions

Our results suggested that the increase of TLR4 expression in CD38^−/−^ mice might be able to promote the production of IFN-*γ*, which contributes to more severe kidney injury in sepsis induced by LPS. Thus, clinically, IFN-*γ* or CD38 can be potential targets for drug development to attenuate sepsis-induced kidney injury.

## Data Availability

The data used to support the findings of this study are available from the corresponding author upon request.
